# The association between a lifestyle score, socioeconomic status, and COVID-19 outcomes within the UK Biobank cohort

**DOI:** 10.1186/s12879-022-07132-9

**Published:** 2022-03-30

**Authors:** Hamish M. E. Foster, Frederick K. Ho, Frances S. Mair, Bhautesh D. Jani, Naveed Sattar, Srinivasa Vittal Katikireddi, Jill P. Pell, Claire L. Niedzwiedz, Claire E. Hastie, Jana J. Anderson, Barbara I. Nicholl, Jason M. R. Gill, Carlos Celis-Morales, Catherine A. O’Donnell

**Affiliations:** 1 Institute of Health and Wellbeing, College of Medical, Veterinary and Life Sciences, University of Glasgow, Glasgow, UK; 2 British Heart Foundation Glasgow Cardiovascular Research Centre, Institute of Cardiovascular and Medical Sciences, University of Glasgow, Glasgow, UK

**Keywords:** COVID-19, Epidemiology, Socioeconomic factors, Lifestyle, Health behaviours

## Abstract

**Background:**

Infection with SARS-CoV-2 virus (COVID-19) impacts disadvantaged groups most. Lifestyle factors are also associated with adverse COVID-19 outcomes. To inform COVID-19 policy and interventions, we explored effect modification of socioeconomic-status (SES) on associations between lifestyle and COVID-19 outcomes.

**Methods:**

Using data from UK-Biobank, a large prospective cohort of 502,536 participants aged 37–73 years recruited between 2006 and 2010, we assigned participants a lifestyle score comprising nine factors. Poisson regression models with penalised splines were used to analyse associations between lifestyle score, deprivation (Townsend), and COVID-19 mortality and severe COVID-19. Associations between each exposure and outcome were examined independently before participants were dichotomised by deprivation to examine exposures jointly. Models were adjusted for sociodemographic/health factors.

**Results:**

Of 343,850 participants (mean age > 60 years) with complete data, 707 (0.21%) died from COVID-19 and 2506 (0.76%) had severe COVID-19. There was evidence of a nonlinear association between lifestyle score and COVID-19 mortality but limited evidence for nonlinearity between lifestyle score and severe COVID-19 and between deprivation and COVID-19 outcomes. Compared with low deprivation, participants in the high deprivation group had higher risk of COVID-19 outcomes across the lifestyle score. There was evidence for an additive interaction between lifestyle score and deprivation. Compared with participants with the healthiest lifestyle score in the low deprivation group, COVID-19 mortality risk ratios (95% CIs) for those with less healthy scores in low versus high deprivation groups were 5.09 (1.39–25.20) and 9.60 (4.70–21.44), respectively. Equivalent figures for severe COVID-19 were 5.17 (2.46–12.01) and 6.02 (4.72–7.71). Alternative SES measures produced similar results.

**Conclusions:**

Unhealthy lifestyles are associated with higher risk of adverse COVID-19, but risks are highest in the most disadvantaged, suggesting an additive influence between SES and lifestyle. COVID-19 policy and interventions should consider both lifestyle and SES. The greatest public health benefit from lifestyle focussed COVID-19 policy and interventions is likely to be seen when greatest support for healthy living is provided to the most disadvantaged groups.

**Supplementary Information:**

The online version contains supplementary material available at 10.1186/s12879-022-07132-9.

## Background

The impact of the coronavirus disease (COVID-19) pandemic has been unequal across societies with consistent evidence of clear social gradients [1–4]. Mechanisms behind these gradients have been attributed to more socioeconomically disadvantaged groups having greater occupational exposure to COVID-19 [5], fewer economic resources to follow protective COVID-19 guidance [6], longer times to diagnosis and clinical management [7], greater non-communicable disease (NCD) and multimorbidity burden [8], and higher prevalence of unhealthy lifestyle factors or health behaviours [9].

The prevalence of combinations of unhealthy lifestyle factors (e.g., smoking, alcohol consumption, and physical inactivity) also follows a socioeconomic status (SES) gradient [10]; and these factors are also associated with severe COVID-19 disease [9]. Indeed, adhering to healthy lifestyle factors such as sufficient sleep and adequate physical activity are cited as ways of reducing the risk of severe COVID-19 [11, 12]. The importance of having a healthy population both in terms of lifestyle and in terms of lifestyle-associated NCDs (e.g., diabetes, obesity, and hypertension) to reduce COVID-19 harms and to withstand future pandemics is clear [13]. However, given the known SES gradient of lifestyle factors (differential exposure), understanding how the risk of COVID-19 varies across SES groups (differential susceptibility), would inform lifestyle policy and interventions that aim to mitigate COVID-19 or future pandemics [14]. This is not least because individual and social resources available to support healthy lifestyles, including perceiving unhealthy lifestyle factors as behavioural choices, also follow a social gradient (differential capacity) [15]. Evidence that SES modifies the association between a comprehensive measure of lifestyle and COVID-19 outcomes would highlight the most vulnerable populations and provide population level targets for increasing support and capacity for healthy living to optimise COVID-19 mitigation efforts [14].

Previous analysis of UK Biobank showed how associations between an extended measurement of unhealthy lifestyle factors—incorporating smoking, excessive alcohol consumption, poor diet, physical inactivity, sleep duration and television viewing time—and all-cause and cardiovascular disease (CVD) mortality was disproportionately stronger in those from more socioeconomically disadvantaged backgrounds [16]. This extended measurement of lifestyle includes both ‘traditional’ and ‘emerging’ lifestyle factors (e.g., sleep duration and television viewing time) and therefore potentially captures wider lifestyle associated risks [17]. Mechanisms behind the disproportionate lifestyle harm are unclear but could include accelerated biological ageing due to higher cumulative risks over the life course in more disadvantaged groups [18, 19]. Similar mechanisms could explain any disproportionate lifestyle harm in terms of COVID-19 outcomes. However, to our knowledge, the influence of both SES and a combination of unhealthy lifestyle factors that includes emerging lifestyle factors on risk of COVID-19 outcomes is unknown. Our aim, therefore, is to investigate the potential effect modification of SES on the association between an extended lifestyle score and adverse COVID-19 outcomes using the UK Biobank population cohort.

We acknowledge that the word ‘lifestyle’ might over-emphasise the role of individual choice for unhealthy behaviours while ignoring the role of wider socioeconomic influences and result in victim blaming [20, 21]. Furthermore, ‘lifestyle medicine’ might have strong but veiled links with alternative medicines that lack evidence [22]. While we recognise this linguistic problem, we use ‘lifestyle factors’ here due to the widespread understanding for their potential to predict adverse health outcomes and to be modified, leading to a reduction or delay in adverse health outcomes [23].

## Methods

### Study design and data collection

Data were from 502,505 participants aged 37–73 years recruited to the UK Biobank population-based cohort study between March 2006 and December 2010 [24]. Baseline demographic, lifestyle, and health data were collected from participants at recruitment via self-administered touch-screen questionnaire and nurse-led interview at 22 assessment centres across England, Scotland, and Wales. A series of measurements were taken by trained staff including height, weight, and blood pressure. Dates of death were obtained from death certificates held by the NHS Information Centre (England and Wales) or the NHS Central Register (Scotland) for all participants. COVID-19 mortality analyses were censored on 28th February 2021 or date of death if death occurred earlier. Participants who died prior to the COVID-19 pandemic (1st March 2020) were excluded. Those who died during the study period from causes other than COVID-19 were regarded as those without the outcome. Data on hospital admissions were obtained from Hospital Episode Statistics (HES; England) and Scottish Morbidity Records (Scotland) and were available up to 31st March 2021. Updated HES data were unavailable for participants who attended baseline assessment centres in Wales and therefore these participants were excluded from analysis of the composite severe COVID-19 outcome.

### Exposures and covariates

All exposure and covariate data were collected at initial baseline assessment (touch-screen questionnaire and nurse-led interview). Baseline assessment was completed by all participants between March 2006 and December 2010.

For the lifestyle exposure, we used a risk score based on that published by Ding et al.[25] which has been used in UK Biobank analysis previously [16]. Briefly, the score is comprised of nine lifestyle factors: smoking status, alcohol intake, physical activity (PA), television viewing time, sleep duration, fruit and vegetable intake, oily fish intake, and red and processed meat intake. Using national guidelines where available, each lifestyle factor was dichotomised into unhealthy and healthy categories. Participants were assigned one point for each unhealthy category (current smoker; alcohol consumed daily or almost daily; physically inactive; ≥ 4 h (h) per day of television viewing time; < 7 h or > 9 h of sleep per day; < 400 g of fruits and vegetables per day; less than one portion of oily fish per week; more than three portions of red meat per week; more than one portion of processed meat per week). Participants' points were summed to create an unweighted score. The minimum score of 0 represents a healthier lifestyle while the maximum score of 9 represents an unhealthier lifestyle. Due to missing data for some variables (walking, moderate, and vigorous physical activity), PA was altered from the previous UK Biobank analysis and instead based on participants’ responses to a single question regarding multiple types of physical activity (walking for pleasure, other exercise, strenuous sports, light or heavy DIY). Individuals were classified as inactive if they did not engage in any of the types of physical activity listed above.

For the SES exposure, three measures were examined: Townsend score (area-level deprivation), annual household income (household-level), and maximum education attainment (individual-level). Townsend scores are derived from data on unemployment, car ownership, household overcrowding, and owner occupation aggregated at postcode area [26]. Townsend scores were assigned to participants based on their address at recruitment and were calculated immediately prior to recruitment using data from the preceding national census data (2001). Higher Townsend scores equate to higher levels of socioeconomic deprivation. Household income (£/year) was self-reported at baseline and categorised as: < 18,000; 18,000–30,999; 31,000–51,999; 52,000–100,000; and > 100,000. Educational attainment, derived from self-reported qualifications at baseline and based on previous UK Biobank analyses using the International Standard Classification of Education [27], was categorised ordinally as: College or University degree; A-levels/AS levels/equivalent (pre-university qualifications); O-levels/GCSEs/equivalent (qualifications taken prior to A or AS-level); CSEs/equivalent (qualifications typically taken at aged 16 years prior to A or AS-level, but aimed at less able pupils than those taking O-levels); None of the above. NVQ/HND/HNC/equivalent (work-based vocational/higher educational qualifications) and ‘Other professional qualifications’ were discounted as it is unclear where these would fit in the hierarchy.

Covariate data included age (recalculated as of 1st March 2020), sex (categorised as male/female) and ethnicity (categorised as White, South Asian, Black, Chinese, Mixed, and Other) were self-reported at baseline. Baseline long-term conditions (LTCs) were based on self-reported physician diagnoses and confirmed at nurse-led interview. LTC count was based on a published list of 43 LTCs and categorised as 0, 1, 2, 3, 4, and ≥ 5 [28].

### Outcomes

We examined two COVID-19 outcomes:

1. COVID-19 mortality (from 1st March 2020 to 28th February 2021), where COVID-19 was given as a contributory cause of death (ICD-10 codes U07.1 or U07.2).
2. composite outcome of severe COVID-19—defined as COVID-19 admission to hospital or death from COVID-19 (from 1st March 2020 to 31st March 2021).

### Statistical analyses

To describe cohort characteristics, we compared participants by lifestyle score category (most healthy, score 0–2; moderately healthy, score 3–5; least healthy, score 6–9) as done previously [16]. We report means and standard deviations for continuous variables and percentages for categorical variables. To analyse the associations between lifestyle score, socioeconomic deprivation and COVID-19 outcomes, we used Poisson regression models with robust standard errors [29]. We specifically estimate the standard errors using a robust method so that the underinflation of variance in using Poisson regression to model binary outcomes can be rectified. Results are reported as risk ratios (RR) with 95% confidence intervals (CIs). Poisson regression models were chosen as they may provide more clinically interpretable results (risk ratios) than logistic regression models (odds ratios) [30]. The association between lifestyle score and COVID-19 may not be linear and therefore we used penalised regression splines to model the association [31]. Penalised thin plate regressions splines have the advantage over cubic splines as a decision over knot location is not required [32].

For our main analysis, we first examined the associations between lifestyle score (continuous) and COVID-19 outcomes and, separately, the association between socioeconomic deprivation (Townsend score; continuous) and COVID-19 outcomes. In these models, the COVID-19 outcomes were dependent variables while the lifestyle score and socioeconomic deprivation were independent variables. To examine the influence of confounders on these associations we incrementally adjusted models for sociodemographic factors and health conditions: Model 0 was unadjusted; Model 1 adjusted for sex, age, and ethnicity; Model 2 as per Model 1 and Townsend score (or lifestyle score where Townsend score was modelled as the main exposure); and Model 3 as per Model 2 and LTC count. Next, we examined for effect modification of deprivation on the association between lifestyle score and COVID-19 outcomes by dichotomising participants by the Townsend score median. Here, the reference group was participants with the healthiest lifestyle (lifestyle score 0) who were also in the low deprivation group (< median Townsend score). For comparison with this reference group, we extracted interval RRs and 95% confidence intervals (CIs) from fully adjusted spline models for the following groups: (1) lifestyle score 0–4 (healthier) and low deprivation; (2) lifestyle score 5–9 (less healthy) and low deprivation; (3) lifestyle score 0–4 and high deprivation; and (4) lifestyle score 5–9 and high deprivation. Two analyses for interactions were conducted. The first was to test for multiplicative interaction where an interaction term was additionally included in the model. Because binary data was modelled on the logarithmic scale, the associations of the independent variables were implicitly assumed to be multiplied (thus ‘multiplicative interaction’). However, the combined effect of the lifestyle score and SES on COVID-19 outcomes might simply be added together rather than being multiplied, and a lack of evidence for multiplicative interaction does not exclude effect modification. Therefore, we also estimated interaction based on the model coefficients to calculate three measures of additive interaction between lifestyle score and Townsend deprivation index on COVID-19 outcomes: relative excess risk due to interaction (RERI), attributable portion due to interaction, and a synergy Index [16, 33]. For this analysis, both lifestyle score and Townsend index were treated as continuous variables and dichotomised by the median, respectively. Additive interaction assumes lifestyle factor and deprivation’s effect are added rather than multiplied. For sensitivity analyses, we repeated all analyses and replaced the area-based Townsend score with each of the alternative SES measures (income and educational attainment) separately. All statistical analysis and graph production was done with R version 4.0.2 with the package ‘mgcv’. Recommendations for strengthening the reporting of observational studies in epidemiology (STROBE) were followed (Additional file 2) [34].

### Role of the funding source

This study used UK Biobank data and was designed, conducted, analysed, and interpreted by the authors who had full access to data in the study. Funding sources are listed in acknowledgements and played no role at any stage of this study. The corresponding author had final responsibility for the decision to submit for publication.

## Results

A flow chart of included participants is shown in Additional file 1: Fig. S1. Of 502,536 participants recruited, 29,307 (5.83%) died prior to the study period and were excluded. Another 129,379 (25.74%) with incomplete lifestyle, demographic, or outcome data were excluded. Comparison of characteristics of those excluded due to missing data with those included is shown in Additional file 1: Table S1. Compared with those with complete data, participants with incomplete data tended to be older, male, from minority ethnic background, be of lower SES, and have less healthy lifestyle factors. Of the 343,850 participants included in mortality analyses, 707 (0.21%) died from COVID-19. Exclusion of participants originally assessed in Wales left 329,274 participants included in severe COVID-19 analyses, of which 2506 (0.76%) had severe COVID-19.

Participant characteristics by categories of lifestyle score are shown in Table 1. Mean age of all participants, recalculated at time of the analysis, was > 60 years in each lifestyle score category. Compared with those with moderately healthy and most healthy lifestyle scores, participants with the least healthy scores were more likely to be younger, male, from Black, Chinese, Mixed or Other ethnic groups, have lower educational attainment, lower income, live in more socioeconomically deprived areas, and have more LTCs. Higher proportions of those with least healthy lifestyle scores died from COVID-19 and had severe COVID-19 compared with those with healthier lifestyle scores (Table 2). Higher proportions of those from more disadvantaged SES groups (more deprived, lower education, or lower income) died from COVID-19 and had severe COVID-19 compared with those in more advantaged groups (Table 2).

**Table 1:** Characteristics of participants by lifestyle score category^a^

	Most healthy (score = 0–2)	Moderate healthy (score = 3–5)	Least healthy (score = 6–9)
Total N	196,380	141,062	6408
Mean (SD) age in March 2020	66.44 (8.03)	65.94 (8.19)	64.95 (8.13)
Male	80,137 (40.8)	72,777 (51.6)	3522 (55.0)
Ethnicity			
White	188,366 (95.9)	133,841 (94.9)	5962 (93.0)
South Asian	3241 (1.7)	2350 (1.7)	97 (1.5)
Black	1914 (1.0)	2338 (1.7)	175 (2.7)
Chinese	460 (0.2)	501 (0.4)	35 (0.5)
Mixed	996 (0.5)	951 (0.7)	59 (0.9)
Others	1403 (0.7)	1081 (0.8)	80 (1.2)
Income, £/year			
Greater than 100,000	14,779 (7.5)	6199 (4.4)	49 (0.8)
52,000 to 100,000	48,652 (24.8)	26,446 (18.7)	560 (8.7)
31,000 to 51,999	54,170 (27.6)	36,571 (25.9)	1188 (18.5)
18,000 to 30,999	46,612 (23.7)	35,916 (25.5)	1657 (25.9)
Less than 18,000	32,167 (16.4)	35,930 (25.5)	2954 (46.1)
Education attainment			
College or University degree	93,224 (47.5)	45,945 (32.6)	955 (14.9)
A levels/AS levels or equivalent	27,011 (13.8)	18,552 (13.2)	634 (9.9)
O levels/GCSEs or equivalent	43,979 (22.4)	37,464 (26.6)	1694 (26.4)
SEs or equivalent	9610 (4.9)	10,753 (7.6)	750 (11.7)
None of the above	22,556 (11.5)	28,348 (20.1)	2375 (37.1)
Mean (SD) deprivation index^b^	− 1.68 (2.84)	− 1.14 (3.13)	0.76 (3.59)
Smoking status			
Never	118,652 (60.4)	72,756 (51.6)	1573 (24.5)
Previous	71,236 (36.3)	45,115 (32.0)	1112 (17.4)
Current	6492 (3.3)	23,191 (16.4)	3723 (58.1)
Consumes alcohol daily or almost daily	10,205 (5.2)	23,011 (16.3)	2602 (40.6)
Physically inactive	21,605 (16.9)	14,404 (18.8)	514 (20.9)
Mean (SD) TV viewing, hours/day	2.24 (1.23)	3.19 (1.66)	4.73 (2.05)
Mean (SD) sleeping duration, hours/day	7.30 (0.87)	6.96 (1.18)	6.49 (1.57)
Sleep duration category			
< 7 h	26,228 (13.4)	51,484 (36.5)	4324 (67.5)
7–9 h	169,074 (86.1)	86,183 (61.1)	1624 (25.3)
> 9 h	1,078 (0.5)	3,395 (2.4)	460 (7.2)
Fruit/vegetable intake < 400 g/day	109,753 (55.9)	124,726 (88.4)	6224 (97.1)
Red meat intake > 3 portions/week	10,622 (5.4)	33,201 (23.5)	3222 (50.3)
Processed meat intake > 1/week	51,880 (26.4)	95,404 (67.6)	5791 (90.4)
Oily fish intake < 1/week	28,568 (14.5)	72,367 (51.3)	5177 (80.8)
Long term condition count			
0	76,779 (39.1)	48,774 (34.6)	1572 (24.5)
1	66,145 (33.7)	46,001 (32.6)	1840 (28.7)
2	33,849 (17.2)	26,733 (19.0)	1433 (22.4)
3	13,254 (6.7)	12,286 (8.7)	846 (13.2)
4	4373 (2.2)	4718 (3.3)	429 (6.7)
5 or more	1980 (1.0)	2550 (1.8)	288 (4.5)

Numbers are n (%) unless otherwise specified

^a^Lifestyle score comprised of nine factors. One point was awarded for each unhealthy factor: current smoker; alcohol consumed daily or almost daily; physically inactive; ≥ 4 h/day of television viewing time; < 7 h or > 9 h of sleep/day; < 400 g of fruits and vegetables/day; less than one portion of oily fish/week; more than three portions of red meat/week; more than one portion of processed meat/week

^b^Townsend derivation index (z-scores) where higher numbers denote higher levels of socioeconomic deprivation

**Table 2:** Number of COVID outcome events by lifestyle score and measures of socioeconomic status

	COVID-19 deaths, n (row %)		Severe COVID-19, n (row %)	
	No	Yes	No	Yes
Lifestyle score				
Most healthy	196,093 (99.9)	287 (0.1)	187,081 (99.4)	1,044 (0.6)
Moderate healthy	140,680 (99.7)	382 (0.3)	133,704 (99.0)	1,310 (1.0)
Least healthy	6,370 (99.4)	38 (0.6)	6,022 (98.2)	113 (1.8)
Townsend deprivation quintile				
1: least deprived	70,884 (99.9)	100 (0.1)	67,231 (99.5)	356 (0.5)
2	69,715 (99.9)	104 (0.1)	66,406 (99.4)	375 (0.6)
3	69,452 (99.8)	129 (0.2)	65,754 (99.4)	418 (0.6)
4	69,535 (99.8)	152 (0.2)	66,225 (99.2)	542 (0.8)
5: most deprived	63,557 (99.7)	222 (0.3)	61,191 (98.7)	776 (1.3)
Household income, £/year				
Greater than 100,000	21,015 (99.9)	12 (0.1)	20,387 (99.7)	71 (0.3)
52,000 to 100,000	75,588 (99.9)	70 (0.1)	72,293 (99.6)	292 (0.4)
31,000 to 51,999	91,815 (99.9)	114 (0.1)	87,310 (99.4)	491 (0.6)
18,000 to 30,999	84,012 (99.8)	173 (0.2)	79,808 (99.3)	600 (0.7)
Less than 18,000	70,713 (99.5)	338 (0.5)	67,009 (98.5)	1013 (1.5)
Education attainment				
College or University degree	139,938 (99.9)	186 (0.1)	13,3802 (99.5)	661 (0.5)
A level or equivalent	46,132 (99.9)	65 (0.1)	44,085 (99.4)	255 (0.6)
O level or equivalent	82,982 (99.8)	155 (0.2)	78,602 (99.3)	588 (0.7)
SEs or equivalent	21,088 (99.9)	25 (0.1)	20,023 (99.3)	148 (0.7)
None of the above	53,003 (99.5)	276 (0.5)	50,295 (98.4)	815 (1.6)

**Fig. 1:**
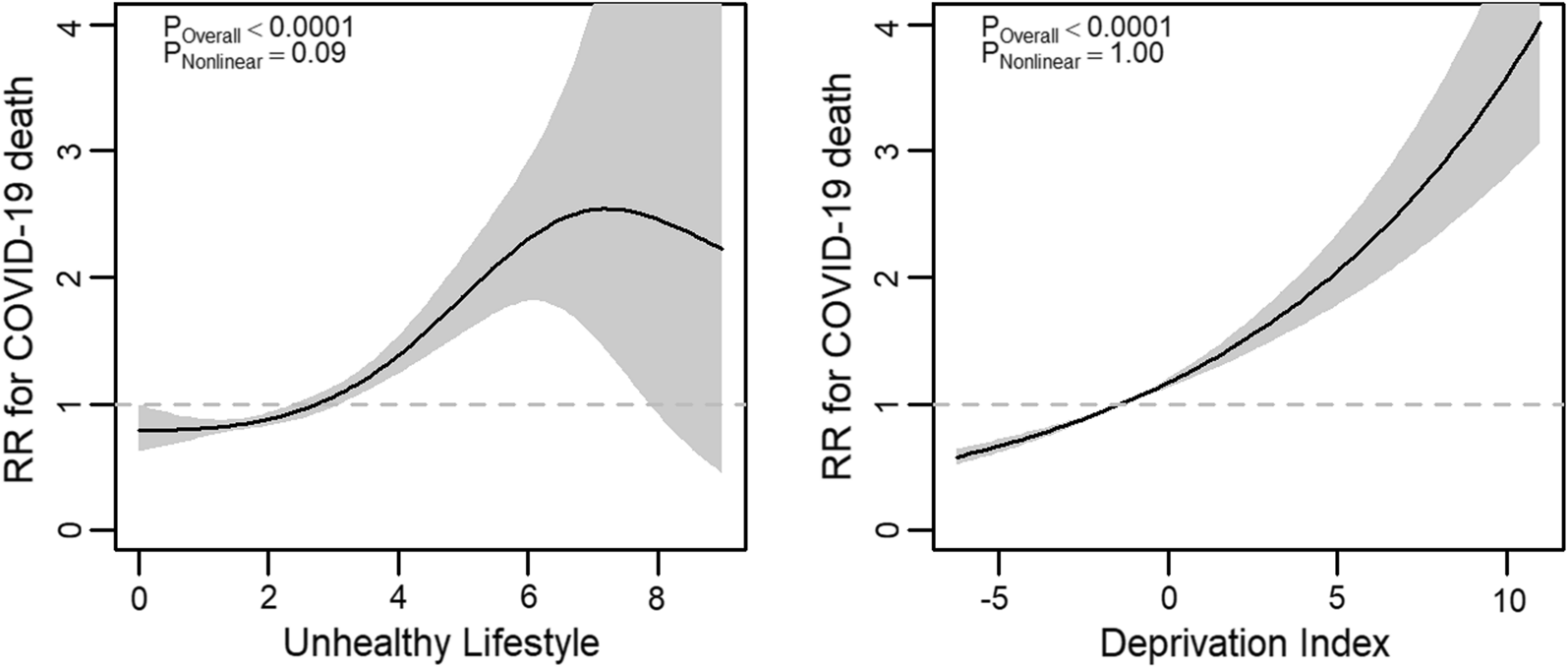
Fully adjusted models examining association between lifestyle score, deprivation, and COVID-19 mortality. *RR* risk ratio; shaded areas show 95% confidence intervals; higher lifestyle score indicates less healthy lifestyle, higher Townsend deprivation index indicates higher socioeconomic deprivation

**Fig. 2:**
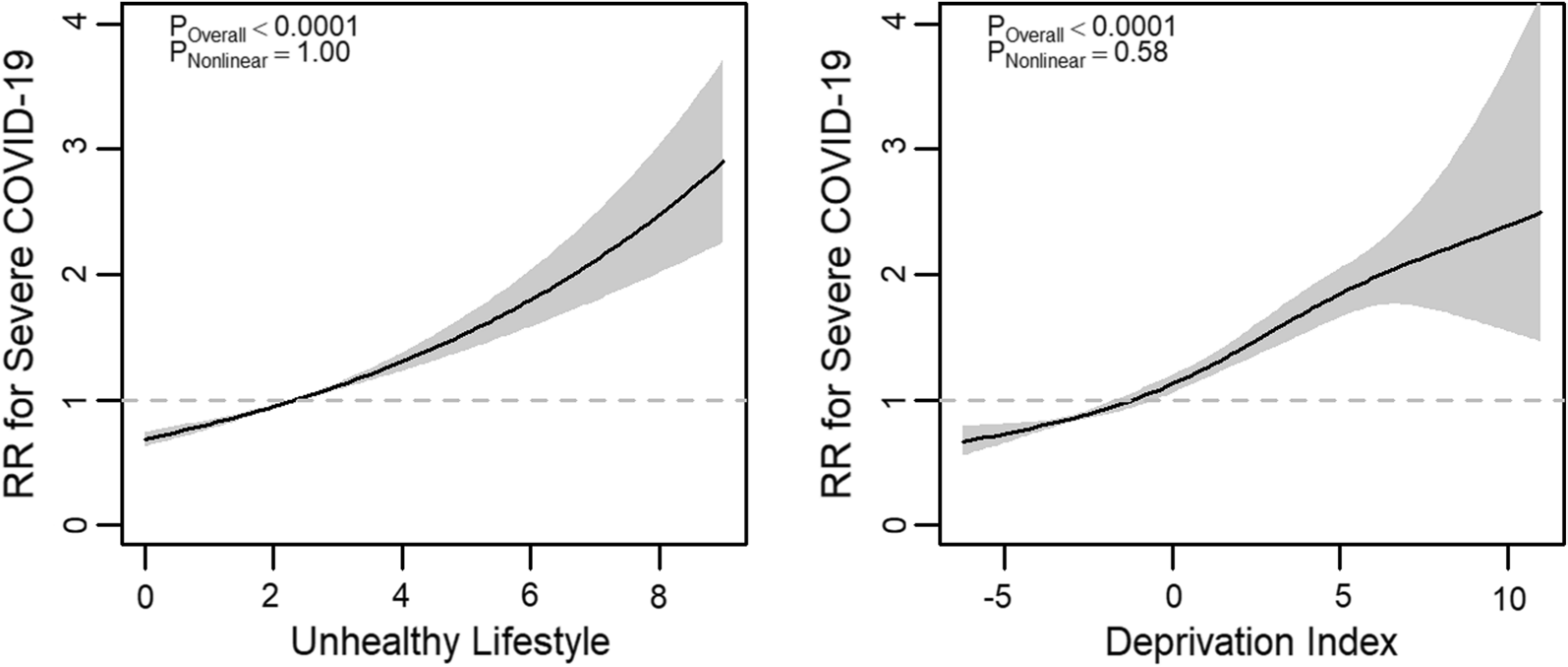
Fully adjusted models examining association between lifestyle score, deprivation, and severe COVID-19. *RR* risk ratio; shaded areas show 95% confidence intervals; higher lifestyle score indicates less healthy lifestyle, higher Townsend deprivation index indicates higher socioeconomic deprivation

In fully adjusted models examining lifestyle score, there was some evidence of a nonlinear association with COVID-19 mortality (Fig. 1) but there was a lack of evidence for a nonlinear association with severe COVID-19 (Fig. 2). Equivalent models examining deprivation lacked evidence for nonlinear associations with both outcomes (Figs. 1, 2). Both an unhealthier lifestyle score and higher levels of deprivation were associated with greater risks of both COVID-19 outcomes. The strength of association between both lifestyle score and deprivation index and both COVID outcomes was similar for both exposures and was stronger (steeper curves) for COVID-19 mortality. However, confidence intervals widened at the unhealthy end of the lifestyle score due to fewer COVID outcome events. Compared with the unadjusted model (Model 0), incremental adjustment for age, sex, and ethnicity (Model 1); deprivation/lifestyle score (Model 2); and then for LTC count (Model 3) minimally attenuated the associations between both lifestyle score and deprivation and both COVID-19 outcomes (Additional file 1: Figs. S2, S3). Substituting Townsend score with either education or income in sensitivity analyses provided similar results (Additional file 1: Figs. S2, S3). There was greater evidence for nonlinearity in the association between income and both outcomes. However, the strength of association between education or income with both COVID-19 outcomes was weaker compared with Townsend score.

**Fig. 3:**
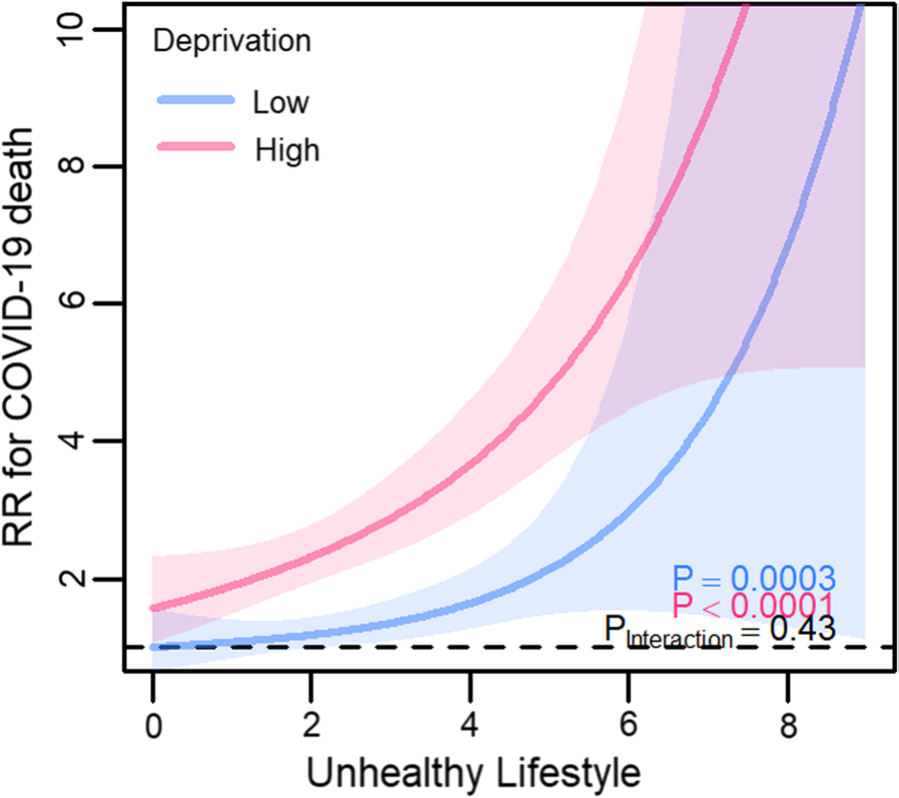
Fully adjusted model examining effect modification of deprivation on the associations between lifestyle score and COVID-19 mortality. *RR* risk ratio; shaded areas show 95% confidence intervals; participants dichotomised into ‘low’ and ‘high’ deprivation groups at median Townsend deprivation index

**Fig. 4:**
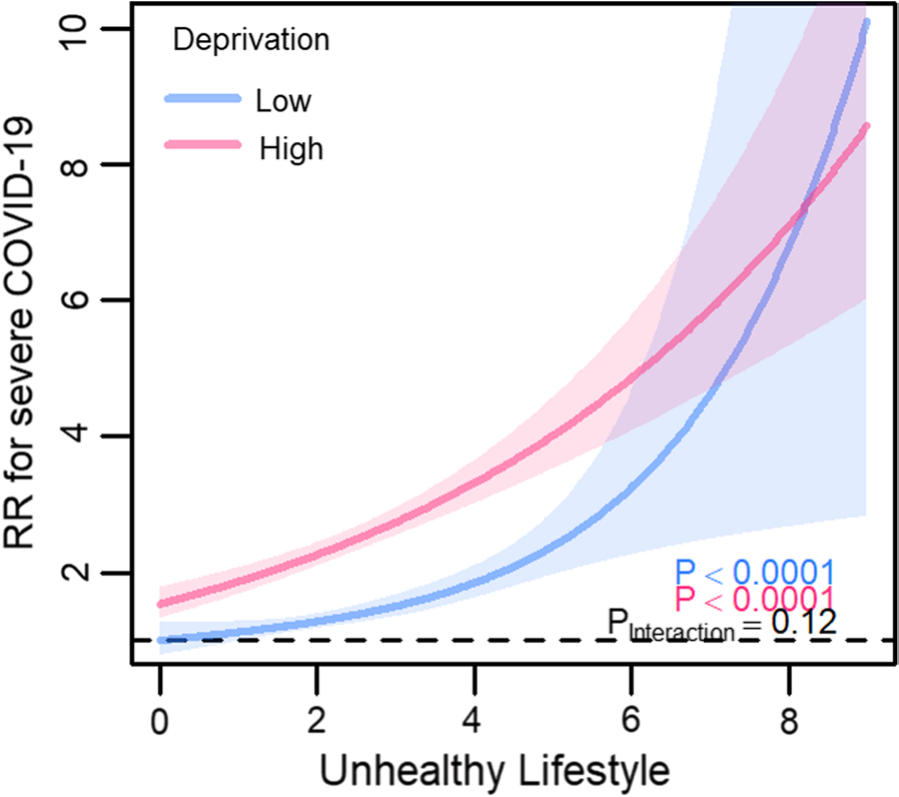
Fully adjusted model examining effect modification of deprivation on the associations between lifestyle score and severe COVID-19. *RR* risk ratio; shaded areas show 95% confidence intervals; participants dichotomised into ‘low’ and ‘high’ deprivation groups at median Townsend deprivation index

Fully adjusted models examining for potential effect modification of deprivation on the associations between lifestyle score and COVID-19 mortality showed that participants in the high deprivation group had a higher risk at each level of lifestyle score compared with those in the less deprived group (Fig. 3). However, confidence intervals were wide with considerable overlap between deprivation groups in those with an unhealthy lifestyle score. When interval RRs (95% CIs) for lifestyle scores between 5 and 9 (less healthy) were extracted from splines, compared with the reference group (participants with lifestyle score 0—‘healthiest’—and also in the low deprivation group), the risk of COVID-19 mortality was 5.09 (1.39–25.20) in the low deprivation group and even higher in those with a similarly less healthy score but in the high deprivation group: 9.60 (4.70–21.44) (Table 3). A similar result was seen for severe COVID-19 but there was some evidence that participants from the low deprivation group appeared to have a higher risk than those in the high deprivation group but only at the very unhealthy extreme of the lifestyle score (i.e., lifestyle score ≥ 8; Fig. 4). However, CIs were wide with complete overlap at the unhealthier end of the lifestyle score (i.e., lifestyle score > 6), with the widest CIs for those in the low deprivation group. Interval RRs (95% CIs) for participants with lifestyle scores between 5 and 9 (less healthy) for severe COVID-19 were 5.17 (2.46–12.01) in the low deprivation group and 6.02 (4.72–7.71) in the high deprivation group (Table 3).

**Table 3:** Interval risk ratios from fully adjusted models examining effect modification of deprivation on the associations between lifestyle score and COVID-19 outcomes

Lifestyle score (LS): deprivation group	Risk ratios (95% confidence intervals)	
	COVID-19 mortality	Severe COVID-19
LS 0: low deprivation	1 (reference)	1 (reference)
LS 0–4 (healthier): low deprivation	1.22 (0.96–1.57)	1.32 (1.18–1.49)
LS 5–9 (less healthy): low deprivation	5.09 (1.39–25.20)	5.17 (2.46–12.01)
LS 0–4 (healthier): high deprivation	2.41 (1.93–3.02)	2.31 (2.12–2.53)
LS 5–9 (less healthy): high deprivation	9.60 (4.70–21.44)	6.02 (4.72–7.71)

There was limited evidence of multiplicative interaction between lifestyle score and Townsend deprivation for both COVID-19 outcomes (P_Interaction_ > 0.05) in all models (Additional file 1: Figs. S4, S5). Similar results were seen in sensitivity analyses with the alternative SES measures (Additional file 1: Figs. S4, S5). However, in unadjusted models, there was some evidence for a multiplicative interaction between income and lifestyle score for COVID-19 mortality (P_Interaction_ = 0.03), but this interaction was attenuated in the fully adjusted model (P_Interaction_ = 0.20). Similarly, in the unadjusted model there was some evidence for a multiplicative interaction between education and lifestyle score for severe COVID-19 (P_Interaction_ = 0.04), but this was attenuated by subsequent adjustment (P_Interaction_ = 0.23 in fully adjusted model). There was evidence of additive interaction with all three measures (RERI, attributable portion and synergy index), indicating additive interaction between lifestyle score and Townsend index for both COVID-19 mortality and severe COVID-19 (Table 4).

**Table 4:** Measures of additive interaction between lifestyle score and Townend index on COVID-19 outcomes

Measure of additive interaction	Estimates (95% confidence intervals)	
	COVID-19 mortality	Severe COVID-19
RERI	0.92 (0.57–1.27)	0.53 (0.29–0.78)
Attributable portion	0.36 (0.23–0.49)	0.22 (0.12–0.31)
Synergy index	2.45 (1.42–4.25)	1.58 (1.22–2.04)

RERI, relative excess risk due to interaction. Where RERI and attributable portion = 0 and synergy index = 1, there is no evidence of additive interaction

## Discussion

### Summary of findings

In the UK Biobank cohort of mid-to-older aged adults, both an unhealthy lifestyle, as estimated by a 9-component lifestyle score, and area-based socioeconomic deprivation have significant associations with COVID-19 mortality and severe COVID-19, even after controlling for potential confounders. Compared with participants from less deprived areas, participants from more deprived areas had generally higher RR for both adverse COVID-19 outcomes at each level of the lifestyle score. Similar results were seen with alternative measures of SES. The highest risks for COVID-19 mortality and severe COVID-19 were seen in the most disadvantaged participants with least healthy lifestyle suggesting an additive influence of both SES and a combination of unhealthy lifestyle factors. This was consistent with formal measures of additive interaction.

### Findings in context with previous research

The need to investigate the modifying effect of SES on harmful exposures in order to guide policy and interventions is well documented, but this type of research is generally lacking [14]. Inequalities in severe COVID-19 across ethnic minority groups, Townsend quartiles, and educational level partially mediated by lifestyle factors (smoking, alcohol intake, and body mass index) have previously been shown in UK Biobank analyses [1]. Further, Woodward et al., also using UK Biobank, identified log-linear associations between Townsend score and COVID-19 mortality via Cox proportional hazard models [3]. This is consistent with the lack of evidence for nonlinear associations identified in our study. Woodward et al. included smoking and related metabolic factors (e.g., BMI and cholesterol) as covariates in their models but did not examine other lifestyle factors like PA, or alcohol intake. Our study has the added value of examining for effect modification of SES on the association between a wide combination of lifestyle factors and COVID-19 outcomes.

The stronger association for the area-based measure of deprivation seen in this study, compared with those for the alternative SES measures of education and income, potentially highlights the role of geographical hot spots for COVID-19 [35]. Further, the Townsend score comprises four components and therefore may capture socioeconomic conditions such as high occupancy housing more completely [36]. Strong associations have been identified between a lifestyle score comprised of four lifestyle factors (smoking, alcohol, physical activity and BMI) and COVID-19 diagnosed in hospital settings [9]. However, that study did not examine associations by SES subgroups, nor did it examine COVID-19 mortality as an outcome. Further, the definition of ‘severe COVID-19’ examined in that study (a positive test result in hospital settings) may have biased estimates as participants may have tested positive with COVID-19 whilst in hospital for other reasons and therefore may not have had severe COVID-19.

The evidence for disproportionate lifestyle-associated COVID-19 risk in less advantaged SES groups on an additive scale reported here differs slightly from similar analyses which identified evidence of a multiplicative interaction between lifestyle and SES for all-cause and CVD mortality [16]. This suggests there may be differences between NCD-linked mortality and COVID-19 or infectious disease mortality due to distinct mechanisms. For example, ageing and cellular senescence might underlie NCD mortality, and this may be accelerated in less advantaged groups [19, 37]. Whereas, acute hyperinflammation, which is more likely to be similar across SES groups, might underlie COVID-19 mortality [38]. In addition, wider social and structural explanations for increased exposure to COVID-19 such as employment type or overcrowded housing may trump or mask any synergistic effect from lifestyle factors [39]. However, the higher risks associated with less advantaged groups at all levels of lifestyle score seen here highlight the need to unpack these mechanisms in order to reduce inequalities associated both with COVID-19, particularly as it becomes endemic, and with future pandemics.

### Strengths and limitations

To our knowledge, this the first study to examine the effect modification of SES on the association between a combination of unhealthy lifestyle factors and COVID-19 outcomes. This study uses a large prospective cohort with rich lifestyle and SES variables and linked routine outcome data which permits construction and analysis of an extended lifestyle score, the use of multiple measures of SES, and linkage to high quality COVID-19 outcome data.

The lifestyle data analysed here was collected 10–15 years prior to the COVID-19 pandemic. Few studies have examined change in multiple lifestyle factors over decades. Both longitudinal and repeated cross-sectional survey data suggest that the lifestyle factors analysed here (smoking, alcohol, physical activity, sedentary behaviours, sleep, and diet), including those in middle-aged adults in the UK, can change over time [40–49]. Despite that and although retirement is associated with lifestyle changes [50], studies suggest only a minority of middle-aged adults make wholescale lifestyle changes [51, 52]. One cohort of 15,708 middle-aged adults in USA showed only 6% made changes from having less than four to having all four healthy lifestyle factors (not smoking, eating at least five fruit and vegetables/day, regular exercise, and maintaining BMI between 18.5–30.0 kg/m^2^) over a 4-year period [52]. In addition, the more healthy lifestyle factors participants reported at the start of that study, the more likely they were to report all 4 healthy lifestyle factors by the end. This finding is pertinent to the nine-factor lifestyle score used in the present study, as it suggests that the ranking of participants by lifestyle remains stable over time (i.e., compared with those with a healthier lifestyle score, those with a less healthy lifestyle score at baseline are less likely to make healthy lifestyle changes and more likely to remain ranked as ‘less healthy’ over time).

Previous studies of UK Biobank also highlight that baseline data can accurately rank participant years later. For example, one study compared dietary baseline data with average daily intakes derived from a more detailed dietary on-line follow up questionnaire [53]. The online follow up questionnaire was completed by subsections of the initial sample (total of 140,080 participants) up to four times over a 16-month period approximately 2–3 years following recruitment. This analysis showed categorisation of participants using the baseline data accurately ranked participants’ average intakes of different food types (e.g., those classified as the highest beef intake category had the highest average beef intake in the online-follow up data). The study also compared dietary data at baseline with repeat assessment data in 20,348 (4.1%) participants at a median of 4.4 years later and showed that oily fish, red meat variables, and processed meat data had substantial agreement (κ values of 0.61–0.8) between the two time points; while equivalent comparison for the four fruit and vegetable items (combined in this study to give total fruit and vegetable consumption) had either moderate or substantial agreement (κ values 0·41–0·60, or 0.61–0.8, respectively). Test–retest reliability of other UK Biobank lifestyle variables has also been assessed: data from 18,905 participants at a mean of 4.3 years apart showed that PA variables and sleep data had lower reliability, but TV viewing time had higher reliability [54]. However, being classified as ‘inactive’ for PA in the present study relied on a combination of responses to a set of PA related questions that was not assessed in that previous study. Therefore, although the lifestyle score at baseline may not accurately reflect lifestyle by the time of follow up in absolute terms, it is more likely to accurately capture relative lifestyle differences between participants. Overall, if there is lifestyle score misclassification, this is likely to introduce regression dilution bias and so estimates reported here may be under-estimates [55].

Similarly, misclassification due to change in SES over time may have also introduced regression dilution bias. However, there is a long-standing lack of social mobility (improvement of SES from more to less disadvantaged) in UK, which, despite some progress with those of more disadvantaged backgrounds achieving more professional qualifications, has been exacerbated by the COVID-19 pandemic [56, 57]. Therefore, although a lack of repeated measurements of SES over time is lacking in the present study, it is likely that, compared to more advantaged groups, those from more disadvantaged SES groups have remained relatively disadvantaged during the 10–15 years between baseline assessment and COVID-19 analysis.

Differential lifestyle score misclassification by SES may have also attenuated our estimates. For example, healthy changes in lifestyle over time are more frequently observed in less disadvantaged SES groups and therefore relative differences in risk between SES groups could be more pronounced than identified here [46, 47, 58, 59]. However, SES differences in lifestyle factor trends remain unclear [60, 61].

UK Biobank had a response rate of 5.5% with proportionally more participants from less disadvantaged SES groups with fewer unhealthy lifestyle factors compared with more nationally representative datasets [62]. In a comparison of the UK Biobank with a nationally representative collection of cohorts, the associations between CVD risk factors and CVD mortality were near identical for some exposures which are similar to those examined here (ever-smoking, less than university education) but higher in UK Biobank for others (physically inactive, current non-drinking of alcohol) [63]. Therefore, as with other comparisons between volunteer-based cohorts and nationally representative samples, the magnitude of associations in less representative samples should be interpreted with caution but the rank ordering of risk of participants is more likely to be generalizable (64). Lifestyle factors can be influenced by health status and co-morbidities and therefore associations identified here may be confounded by poor health and pre-existing illness. The findings from this cohort of mid-to-older aged adults may not be generalisable to other age groups, with younger age groups less likely to die or have severe illness from most diseases. Nevertheless, it is well recognised that, even from early life, there is higher risk of nearly all adverse health outcomes due to both unhealthy lifestyle factors and less advantaged SES and therefore both lifestyle and SES may also influence adverse COVID-19 outcomes in other age groups albeit to greater or lesser degree. Exclusion of 25.74% of participants with incomplete data may have attenuated our estimates and therefore estimates in the general population may be higher. Those with missing data tended to be in conventionally higher risk groups: older, male, more disadvantaged SES, and more unhealthy lifestyle factors (Additional file 1: Table S1). The lifestyle score used here does not allow ascertainment of which factors contribute the most risk, but this would be an important question for future research. Our analysis considered those who died from non-COVID-19 causes during the study period as those without the outcome, and because people with more unhealthy lifestyle factors are at higher risk of all-cause deaths, this may have attenuated the associations between lifestyle and COVID-19 seen here. Finally, observational evidence is always limited by potential residual confounding.

The findings reported here have two important implications. First, a less healthy lifestyle is associated with an increased risk of poor COVID-19 outcomes, suggesting that everyone, regardless of SES, could benefit from support to improve their lifestyle. Second, because the strongest associations between less healthy lifestyles and poor COVID-19 outcomes was found in more disadvantaged groups, both policy and interventions to mitigate against COVID-19 should consider both a combination of lifestyle factors and SES. Therefore, benefit from lifestyle focussed COVID-19 policy and interventions is likely to be greatest when support for healthy living is optimised in the most disadvantaged groups.

## Conclusions

Both a wide combination of unhealthy lifestyle factors and multiple measures of more disadvantaged SES are associated with higher risk of severe COVID-19 outcomes. The additive influence of both these factors further increased the risks for COVID-19 mortality and severe COVID-19 disease. While improving lifestyle could lower everyone’s risk of adverse COVID-19 outcomes, greater support for those in areas of social deprivation would likely bring further public health benefit.

## Supplementary Information

**Additional file 1: Fig. S1**. Flowchart for participants included in analyses. **Fig. S2**. Models of the associations between main exposures (lifestyle score and SES measures) and COVID-19 mortality. **Fig. S3**. Models of the associations between main exposures (lifestyle score and SES measures) and severe COVID-19. **Fig. S4**. Models examining effect modification of SES on the association between lifestyle score and COVID-19 mortality. **Fig. S5**. Models examining effect modification of SES on the association between lifestyle score and severe COVID-19. **Table S1**. Comparison of characteristics of participants with missing and complete data.

**Additional file 2**: STROBE checklist.

## Data Availability

UK Biobank data is not publicly available. However, subject to successful application, all bona fide researchers can access UK Biobank data via www.ukbiobank.ac.uk. Syntax for the generation of derived variables and analysis used in this study will be submitted to UK Biobank for record.

## Abbreviations

A-level: General Certificate of Education Advanced Level
AS-level: Advanced Subsidiary level
CI: Confidence interval
COVID-19: Coronavirus disease due to SARS-CoV-2 virus
CSE: Certificate of Secondary Education
CVD: Cardiovascular disease
GCSE: General Certificate of Secondary Education
HES: Hospital Episode Statistics
HND/HNC: Higher National Diploma/Certificate
LTC: Long term condition
NCD: Noncommunicable disease
NVQ: National Vocational Qualification
O-level: General Certificate of Education: Ordinary Level
RERI: Relative excess risk due to interaction
RR: Risk ratio
SES: Socioeconomic status
UK: United Kingdom

## References

[1] Niedzwiedz CL, O'Donnell CA, Jani BD, Demou E, Ho FK, Celis-Morales C (2020). Ethnic and socioeconomic differences in SARS-CoV-2 infection: prospective cohort study using UK Biobank. BMC Med.

[2] Ho FK, Celis-Morales CA, Gray SR, Katikireddi SV, Niedzwiedz CL, Hastie C (2020). Modifiable and non-modifiable risk factors for COVID-19, and comparison to risk factors for influenza and pneumonia: results from a UK Biobank prospective cohort study. BMJ Open.

[3] Woodward M, Peters SAE, Harris K (2021). Social deprivation as a risk factor for COVID-19 mortality among women and men in the UK Biobank: nature of risk and context suggests that social interventions are essential to mitigate the effects of future pandemics. J Epidemiol Community Health.

[4] Maness SB, Merrell L, Thompson EL, Griner SB, Kline N, Wheldon C (2021). Social determinants of health and health disparities: COVID-19 exposures and mortality among African American People in the United States. Public Health Rep.

[5] Mutambudzi M, Niedwiedz C, Macdonald EB, Leyland A, Mair F, Anderson J (2021). Occupation and risk of severe COVID-19: prospective cohort study of 120 075 UK Biobank participants. Occup Environ Med.

[6] Smith LE, Potts HWW, Amlot R, Fear NT, Michie S, Rubin GJ (2021). Adherence to the test, trace, and isolate system in the UK: results from 37 nationally representative surveys. BMJ.

[7] Wu Y, Yan X, Zhao S, Wang J, Ran J, Dong D (2020). Association of time to diagnosis with socioeconomic position and geographical accessibility to healthcare among symptomatic COVID-19 patients: a retrospective study in Hong Kong. Health Place.

[8] McQueenie R, Foster HME, Jani BD, Katikireddi SV, Sattar N, Pell JP (2020). Multimorbidity, polypharmacy, and COVID-19 infection within the UK Biobank cohort. PLoS ONE.

[9] Hamer M, Kivimaki M, Gale CR, Batty GD (2020). Lifestyle risk factors, inflammatory mechanisms, and COVID-19 hospitalization: a community-based cohort study of 387,109 adults in UK. Brain Behav Immun.

[10] Meader N, King K, Moe-Byrne T, Wright K, Graham H, Petticrew M (2016). A systematic review on the clustering and co-occurrence of multiple risk behaviours. BMC Public Health.

[11] Sallis R, Young DR, Tartof SY, Sallis JF, Sall J, Li QW (2021). Physical inactivity is associated with a higher risk for severe COVID-19 outcomes: a study in 48 440 adult patients. Brit J Sport Med..

[12] Lange KW, Nakamura Y (2020). Lifestyle factors in the prevention of COVID-19. Glob Health J.

[13] Lavie CJ, Sanchis-Gomar F, Arena R (2021). Fit is it in COVID-19, future pandemics, and overall healthy living. Mayo Clin Proc.

[14] Diderichsen F, Hallqvist J, Whitehead M (2019). Differential vulnerability and susceptibility: how to make use of recent development in our understanding of mediation and interaction to tackle health inequalities. Int J Epidemiol.

[15] Smith KE, Anderson R (2018). Understanding lay perspectives on socioeconomic health inequalities in Britain: a meta-ethnography. Sociol Health Illn.

[16] Foster HME, Celis-Morales CA, Nicholl BI, Petermann-Rocha F, Pell JP, Gill JMR (2018). The effect of socioeconomic deprivation on the association between an extended measurement of unhealthy lifestyle factors and health outcomes: a prospective analysis of the UK Biobank cohort. Lancet Public Health.

[17] Krokstad S, Ding D, Grunseit AC, Sund ER, Holmen TL, Rangul V, et al. Multiple lifestyle behaviours and mortality, findings from a large population-based Norwegian cohort study—the HUNT Study. BMC Public Health. 2017;17. 10.1186/s12889-016-3993-x PMC5223537 28068991

[18] Belsky DW, Caspi A, Cohen HJ, Kraus WE, Ramrakha S, Poulton R (2017). Impact of early personal-history characteristics on the Pace of Aging: implications for clinical trials of therapies to slow aging and extend healthspan. Aging Cell.

[19] Fiorito G, Polidoro S, Dugue PA, Kivimaki M, Ponzi E, Matullo G (2017). Social adversity and epigenetic aging: a multi-cohort study on socioeconomic differences in peripheral blood DNA methylation. Sci Rep.

[20] Richards H, Reid M, Watt G (2003). Victim-blaming revisited: a qualitative study of beliefs about illness causation, and responses to chest pain. Fam Pract.

[21] Katikireddi SV, Higgins M, Smith KE, Williams G (2013). Health inequalities: the need to move beyond bad behaviours. J Epidemiol Community Health.

[22] Nunan D, Blane DN, McCartney M (2021). Exemplary medical care or Trojan horse? An analysis of the 'lifestyle medicine' movement. Br J Gen Pract.

[23] Frazer K, Callinan JE, McHugh J, van Baarsel S, Clarke A, Doherty K (2016). Legislative smoking bans for reducing harms from secondhand smoke exposure, smoking prevalence and tobacco consumption. Cochrane Database Syst Rev.

[24] Sudlow C, Gallacher J, Allen N, Beral V, Burton P, Danesh J (2015). UK biobank: an open access resource for identifying the causes of a wide range of complex diseases of middle and old age. PLoS Med.

[25] Ding D, Rogers K, van der Ploeg H, Stamatakis E, Bauman AE (2015). Traditional and emerging lifestyle risk behaviors and all-cause mortality in middle-aged and older adults: evidence from a large population-based Australian Cohort. PLoS Med.

[26] Townsend P, Philimore P, Beattie A. Health and deprivation: inequality and the North: Croom Helm; 1988.

[27] Hagenaars SP, Gale CR, Deary IJ, Harris SE (2017). Cognitive ability and physical health: a Mendelian randomization study. Sci Rep.

[28] Jani BD, Hanlon P, Nicholl BI, McQueenie R, Gallacher KI, Lee D (2019). Relationship between multimorbidity, demographic factors and mortality: findings from the UK Biobank cohort. BMC Med.

[29] Zou G (2004). A modified Poisson regression approach to prospective studies with binary data. Am J Epidemiol.

[30] A'Court C, Stevens R, Heneghan C (2012). Against all odds? Improving the understanding of risk reporting. Br J Gen Pract.

[31] Wood SN (2003). Thin plate regression splines. J Roy Stat Soc B.

[32] Wood SN, Pya N, Safken B (2016). Smoothing parameter and model selection for general smooth models. J Am Stat Assoc.

[33] Andersson T, Alfredsson L, Kallberg H, Zdravkovic S, Ahlbom A (2005). Calculating measures of biological interaction. Eur J Epidemiol.

[34] von Elm E, Altman DG, Egger M, Pocock SJ, Gøtzsche PC, Vandenbroucke JP (2008). STROBE initiative. The strengthening the reporting of observational studies in epidemiology (STROBE) statement: guidelines for reporting observational studies. J Clin Epidemiol.

[35] Public Health England—COVID-19: review of disparities in risks and outcomes. 2020.

[36] Suleman M, Sonthalia S, Webb C, Tinson A, Kane M, Bunbury S, Finch D, Bibby J. The Health Foundation: Unequal pandemic, fairer recovery—the COVID-19 impact inquiry report. The Health Foundation; 2021.

[37] Lopez-Otin C, Blasco MA, Partridge L, Serrano M, Kroemer G (2013). The hallmarks of aging. Cell.

[38] Merad M, Martin JC (2020). Pathological inflammation in patients with COVID-19: a key role for monocytes and macrophages. Nat Rev Immunol.

[39] Patel JA, Nielsen FBH, Badiani AA, Assi S, Unadkat VA, Patel B (2020). Poverty, inequality and COVID-19: the forgotten vulnerable. Public Health.

[40] Adult smoking habits in the UK: 2019. Statistical bulletin. Office for National Statistics UK. 2020.

[41] Adult drinking habits in Great Britain: 2017. Statistical bulletin. Office for National Statistics UK. 2018.

[42] Active Lives Adult Survey, May 2020/21 Report. Sport England. 2021.

[43] Ohayon MM, Carskadon MA, Guilleminault C, Vitiello MV (2004). Meta-analysis of quantitative sleep parameters from childhood to old age in healthy individuals: developing normative sleep values across the human lifespan. Sleep.

[44] Lopez-Valenciano A, Mayo X, Liguori G, Copeland RJ, Lamb M, Jimenez A. Changes in sedentary behaviour in European Union adults between 2002 and 2017. BMC Public Health. 2020;20(1). 10.1186/s12889-020-09293-1 PMC7448983 32843022

[45] Reinders I, van Schoor NM, Deeg DJH, Huisman M, Visser M (2018). Trends in lifestyle among three cohorts of adults aged 55–64 years in 1992/1993, 2002/2003 and 2012/2013. Eur J Public Health.

[46] Rehm CD, Penalvo JL, Afshin A, Mozaffarian D (2016). Dietary Intake Among US Adults, 1999–2012. Jama-J Am Med Assoc.

[47] Dinnissen CS, Ocke MC, Buurma-Rethans EJM, van Rossum CTM (2021). Dietary changes among adults in The Netherlands in the period 2007–2010 and 2012–2016. Results from two cross-sectional national food consumption surveys. Nutrients.

[48] National Diet and Nutrition Survey Rolling programme Years 9 to 11 (2016/2017 to 2018/2019). Public Health England. 2020.

[49] Hulsegge G, Looman M, Smit HA, Daviglus ML, van der Schouw YT, Verschuren WM. Lifestyle changes in young adulthood and middle age and risk of cardiovascular disease and all-cause mortality: the Doetinchem Cohort Study. J Am Heart Assoc. 2016;5(1). 10.1161/JAHA.115.002432 PMC4859361 26764411

[50] Godfrey A, Lord S, Galna B, Mathers JC, Burn DJ, Rochester L (2014). The association between retirement and age on physical activity in older adults. Age Ageing.

[51] White J, Greene G, Kivimaki M, Batty GD (2018). Association between changes in lifestyle and all-cause mortality: the Health and Lifestyle Survey. J Epidemiol Commun H.

[52] King DE, Mainous AG, Geesey ME (2007). Turning back the clock: adopting a healthy lifestyle in middle age. Am J Med.

[53] Bradbury KE, Young HJ, Guo WJ, Key TJ. Dietary assessment in UK Biobank: an evaluation of the performance of the touchscreen dietary questionnaire. J Nutr Sci. 2018;7. 10.1017/jns.2017.66 PMC5799609 29430297

[54] Pearce M, Strain T, Kim Y, Sharp SJ, Westgate K, Wijndaele K (2020). Estimating physical activity from self-reported behaviours in large-scale population studies using network harmonisation: findings from UK Biobank and associations with disease outcomes. Int J Behav Nutr Phys Act.

[55] Hutcheon JA, Chiolero A, Hanley JA (2010). Random measurement error and regression dilution bias. BMJ.

[56] State of the Nation 2016: Social Mobility in Great Britain. The Social Mobility Commission. 2016.

[57] State of the nation 2021: Social mobility and the pandemic. The Social Mobility Commission. 2021.

[58] Griffin A, Roselli T, Clemens SL (2020). Trends in total physical activity time, walking, and vigorous physical activity time in Queensland adults from 2004–2018. J Phys Act Health.

[59] Taylor AW, Dal Grande E, Wu J, Shi Z, Campostrini S (2014). Ten-year trends in major lifestyle risk factors using an ongoing population surveillance system in Australia. Popul Health Metr.

[60] Galobardes B, Costanza MC, Bernstein MS, Delhumeau C, Morabia A (2003). Trends in risk factors for lifestyle-related diseases by socioeconomic position in Geneva, Switzerland, 1993–2000: health inequalities persist. Am J Public Health.

[61] Kim YJ, Lee JS, Park J, Choi DS, Kim DM, Lee KH (2017). Trends in socioeconomic inequalities in five major risk factors for cardiovascular disease in the Korean population: a cross-sectional study using data from the Korea National Health and Nutrition Examination Survey, 2001–2014. BMJ Open.

[62] Fry A, Littlejohns TJ, Sudlow C, Doherty N, Adamska L, Sprosen T (2017). Comparison of sociodemographic and health-related characteristics of UK Biobank participants with those of the general population. Am J Epidemiol.

[63] Batty GD, Gale CR, Kivimaki M, Deary IJ, Bell S (2020). Comparison of risk factor associations in UK Biobank against representative, general population based studies with conventional response rates: prospective cohort study and individual participant meta-analysis. BMJ.

[64] Liao YL, McGee DL, Cooper RS, Sutkowski MBE (1999). How generalizable are coronary risk prediction models? Comparison of Framingham and two national cohorts. Am Heart J.

