# Supplementary material for: The association between a lifestyle score, socioeconomic status, and COVID-19 outcomes within the UK Biobank cohort
Source: BMC Infect Dis. 2022 Mar 30;22:273. doi: 10.1186/s12879-022-07132-9 (PMC8964028; doi:10.1186/s12879-022-07132-9)
Supplement: Supplementary file 1 — Additional file 1: Fig. S1. Flowchart for participants included in analyses. Fig. S2. Models of the associations between main exposures (lifestyle score and SES measures) and COVID-19 mortality. Fig. S3. Models of the associations between main exposures (lifestyle score and SES measures) and severe COVID-19. Fig. S4. Models examining effect modification of SES on the association between lifestyle score and COVID-19 mortality. Fig. S5. Models examining effect modification of SES on the association between lifestyle score and severe COVID-19. Table S1. Comparison of characteristics of participants with missing and complete data. [file 12879_2022_7132_MOESM1_ESM.docx]

Supplementary Material

Supplementary Figure 1. Flowchart for participants included in analyses

502,536 UK Biobank participants

473,229 alive at the start of the COVID-19 pandemic

Excluded 29,307 who died prior to the COVID-19 pandemic

N=343,850
for the analysis of COVID-19 death

Excluded 129,379 who had missing sociodemographic, lifestyle, or outcome data

N=329,274
for the analysis of severe COVID-19

Excluded 14,576 who attended assessment centres in Wales

Supplementary Table 1. Comparison of characteristics of participants with missing and complete data

|  | Missing | Complete data | P |
| --- | --- | --- | --- |
| Mean (SD) age in March 2020 | 68.49 (7.83) | 66.21 (8.11) | < 0.0001 |
| Male | 55235 (42.7) | 156436 (45.5) | < 0.0001 |
| Ethnicity |  |  | < 0.0001 |
| White | 116452 (91.8) | 328169 (95.4) |  |
| South Asian | 3800 (3.0) | 5688 (1.7) |  |
| Black | 3352 (2.6) | 4427 (1.3) |  |
| Chinese | 536 (0.4) | 996 (0.3) |  |
| Mixed | 840 (0.7) | 2006 (0.6) |  |
| Others | 1814 (1.4) | 2564 (0.7) |  |
| Education attainment |  |  | < 0.0001 |
| College or University degree | 14370 (11.5) | 140124 (40.8) |  |
| A levels/AS levels or equivalent | 6558 (5.2) | 46197 (13.4) |  |
| O levels/GCSEs or equivalent | 16596 (13.3) | 83137 (24.2) |  |
| SEs or equivalent | 4745 (3.8) | 21113 (6.1) |  |
| None of the above | 23220 (18.6) | 53279 (15.5) |  |
| Income, £/year |  |  | < 0.0001 |
| Greater than 100,000 | 1344 (1.0) | 21027 (6.1) |  |
| 52,000 to 100,000 | 8270 (6.4) | 75658 (22.0) |  |
| 31,000 to 51,999 | 14491 (11.2) | 91929 (26.7) |  |
| 18,000 to 30,999 | 17296 (13.4) | 84185 (24.5) |  |
| Less than 18,000 | 16476 (12.7) | 71051 (20.7) |  |
| Missing | 71502 (55.3) | 0 (0.0) |  |
| Mean (SD) deprivation index | -1.11 (3.23) | -1.41 (3.01) | < 0.0001 |
| Smoking status |  |  | < 0.0001 |
| Never | 58163 (53.4) | 196380 (57.1) |  |
| Previous | 48112 (44.1) | 141062 (41.0) |  |
| Current | 2708 (2.5) | 6408 (1.9) |  |
| Smoking status |  |  | < 0.0001 |
| Never | 69541 (54.9) | 192981 (56.1) |  |
| Previous | 43361 (34.2) | 117463 (34.2) |  |
| Current | 13795 (10.9) | 33406 (9.7) |  |
| Consumed alcohol daily or almost daily | 18182 (14.2) | 35818 (10.4) | < 0.0001 |
| Physically inactive | 10949 (15.7) | 36523 (17.6) | < 0.0001 |
| Mean (SD) TV viewing, hours/day | 3.02 (1.65) | 2.68 (1.54) | < 0.0001 |
| Mean (SD) sleeping duration, hours/day | 7.16 (1.19) | 7.15 (1.04) | 0.005 |
| Sleep duration |  |  | < 0.0001 |
| < 7 hours | 33476 (26.7) | 82036 (23.9) |  |
| 7-9 hours | 89015 (70.9) | 256881 (74.7) |  |
| > 9 hours | 3052 (2.4) | 4933 (1.4) |  |
| Fruit/vegetable intake <400g/day | 88968 (68.8) | 240703 (70.0) | < 0.0001 |
| Red meat intake >3 portions/week | 19808 (15.3) | 47045 (13.7) | < 0.0001 |
| Process meat intake > once a week | 54368 (42.0) | 153075 (44.5) | < 0.0001 |
| Oily fish intake < once a week | 40286 (31.1) | 106112 (30.9) | 0.07 |
| Morbidity count |  |  | < 0.0001 |
| 0 | 41182 (31.8) | 127125 (37.0) |  |
| 1 | 41926 (32.4) | 113986 (33.1) |  |
| 2 | 26213 (20.3) | 62015 (18.0) |  |
| 3 | 12259 (9.5) | 26386 (7.7) |  |
| 4 | 4916 (3.8) | 9520 (2.8) |  |
| 5+ | 2882 (2.2) | 4818 (1.4) |  |
| Numbers are n (%) unless otherwise specified |  |  |  |

Supplementary Figure 2. Models of the associations between main exposures (lifestyle score and SES measures) and COVID-19 mortality


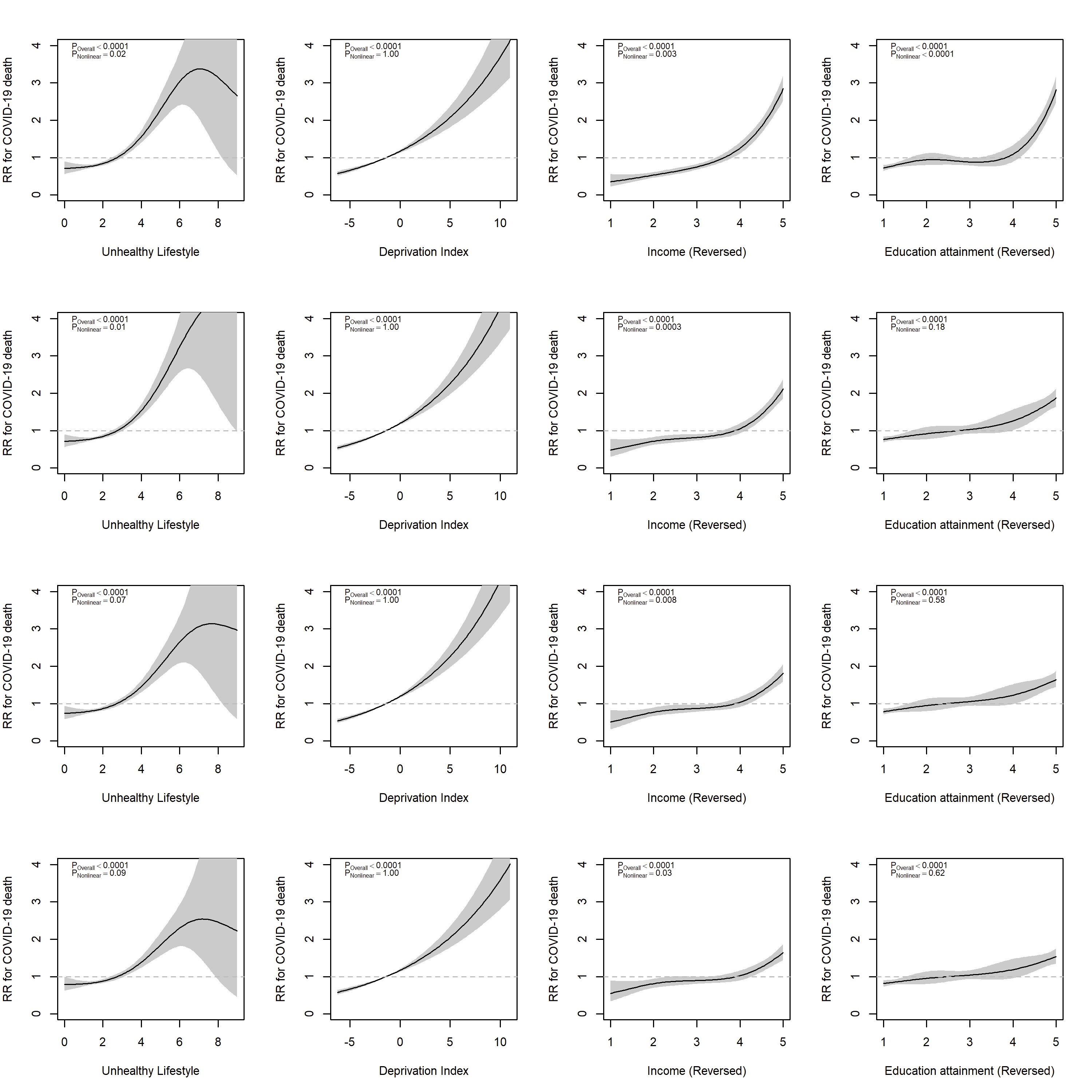


Model 0

Model 1

Model 2

Model 3

Incremental model adjustment:

Model 0 - unadjusted

Model 1 - adjusted for age, sex, and ethnicity

Model 2 - as Model 1 + deprivation/lifestyle score Model 3 - as Model 2 + LTC count


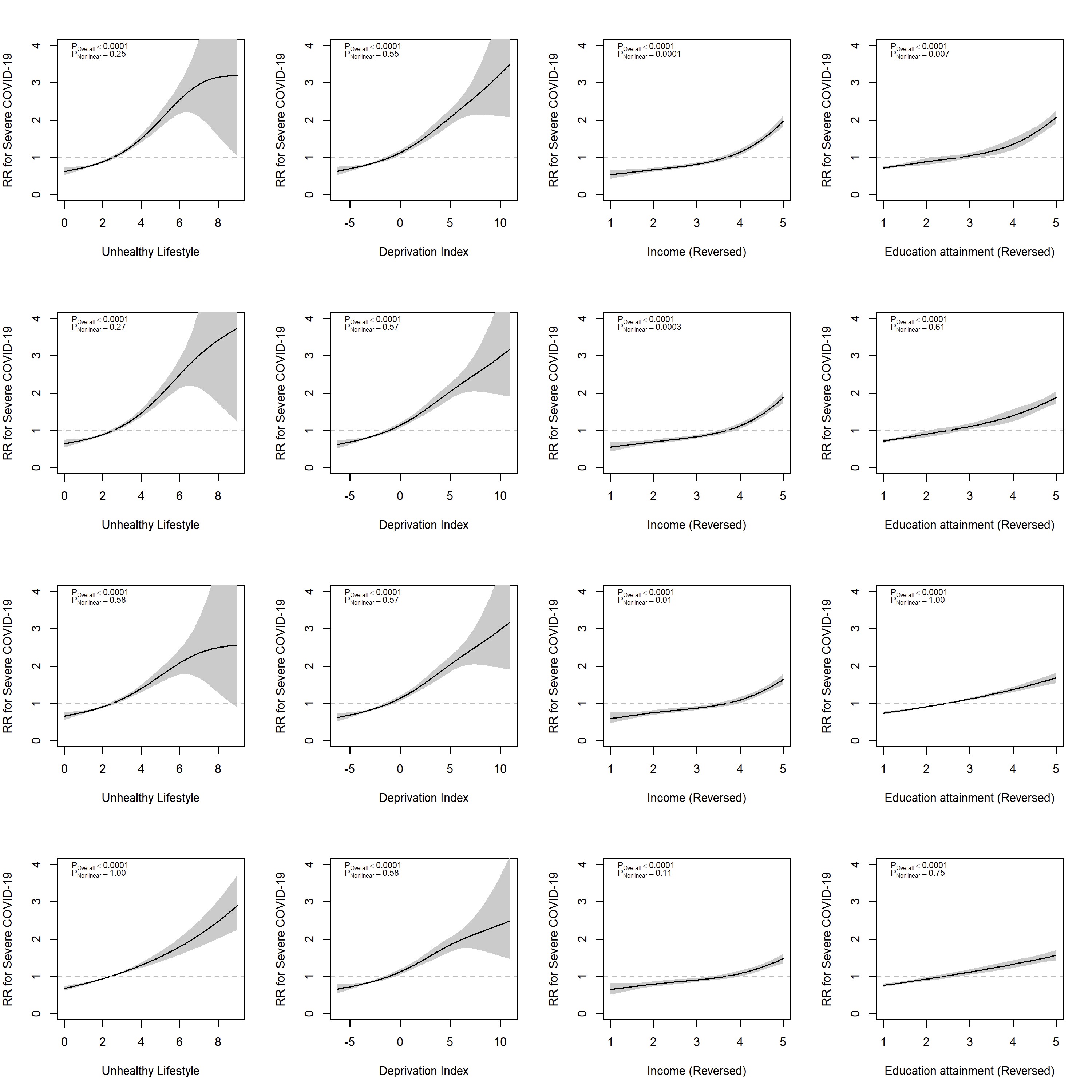


Supplementary Figure 3. Models of the associations between main exposures (lifestyle score and SES measures) and severe
COVID-19.

Model 0

Model 1

Model 2

Model 3

Incremental model adjustment:

Model 0 - unadjusted

Model 1 - adjusted for age, sex, and ethnicity

Model 2 - as Model 1 + deprivation/lifestyle score Model 3 - as Model 2 + LTC count


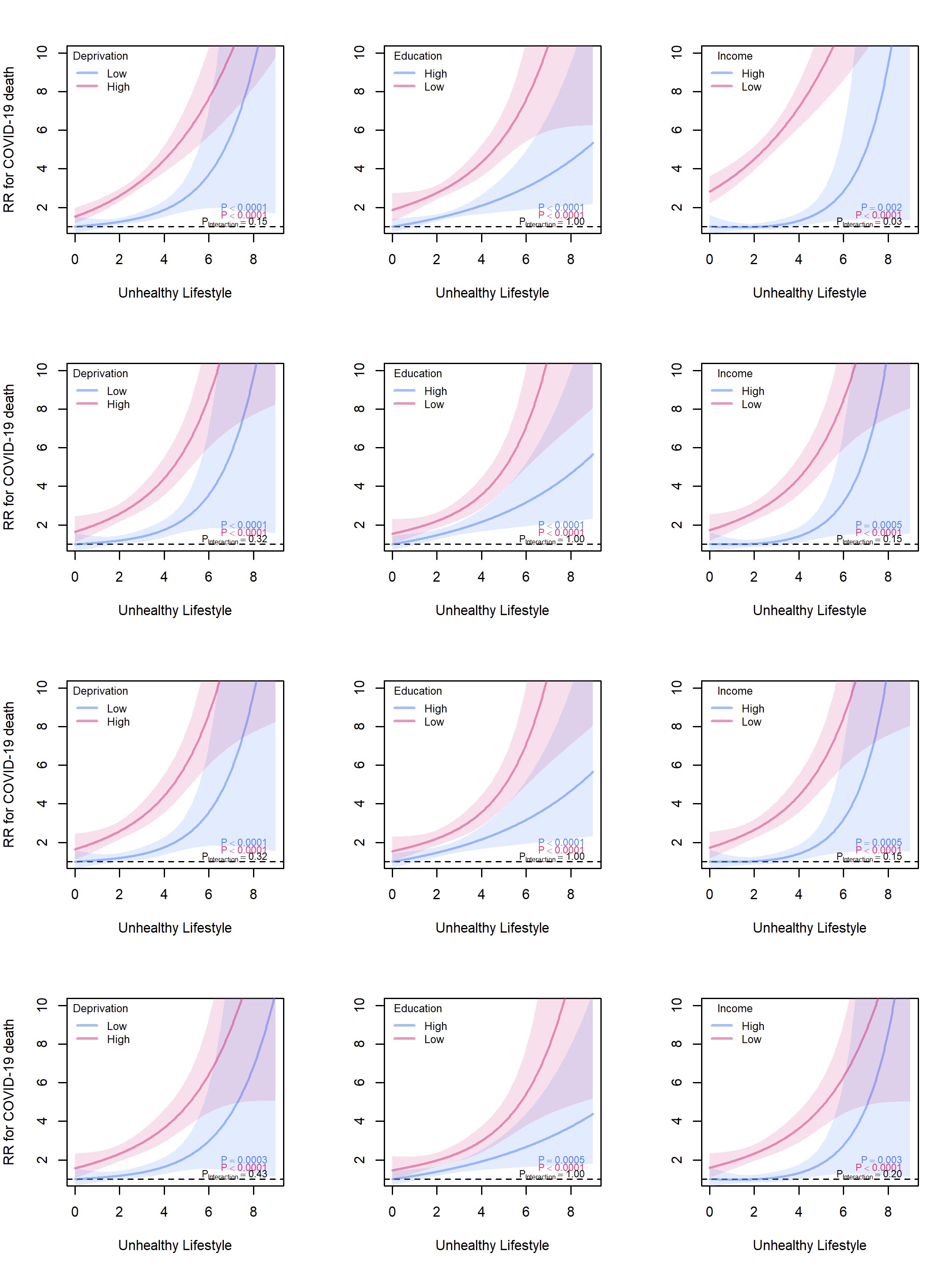


Supplementary Figure 4. Models examining effect modification of SES on the association between lifestyle score and COVID-19 mortality

Model 0

Model 1

Model 2

Model 3

Incremental model adjustment:

Model 0 - unadjusted

Model 1 - adjusted for age, sex, and ethnicity

Model 2 - as Model 1 + deprivation/lifestyle score Model 3 - as Model 2 + LTC count


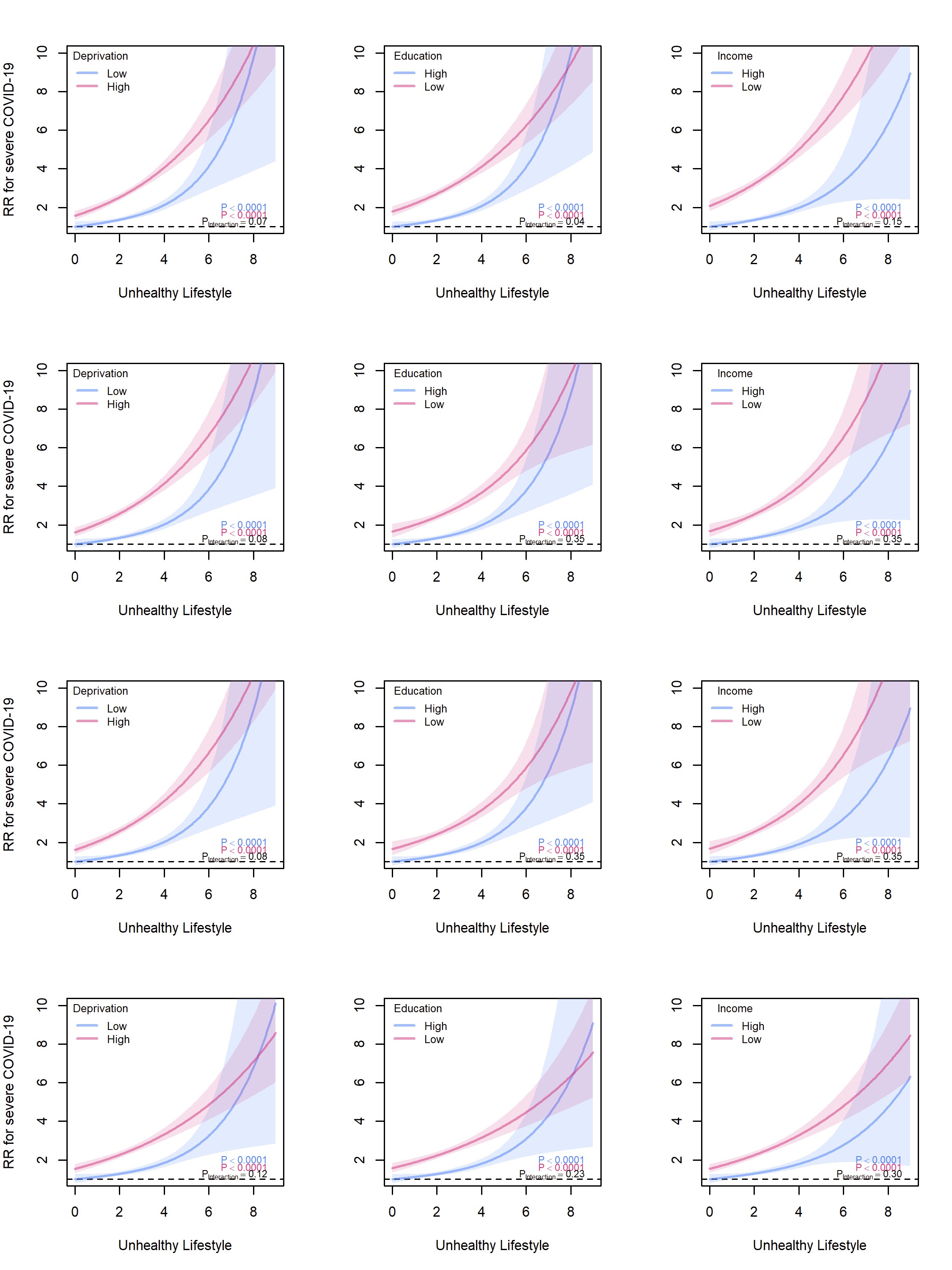


Supplementary Figure 5. Models examining effect modification of SES on the association between lifestyle score and severe COVID-19

Model 0

Model 1

Model 2

Model 3

Incremental model adjustment:

Model 0 - unadjusted

Model 1 - adjusted for age, sex, and ethnicity

Model 2 - as Model 1 + deprivation/lifestyle score Model 3 - as Model 2 + LTC count
